# Electromagnetic Nanoparticles for Sensing and Medical Diagnostic Applications

**DOI:** 10.3390/ma11040603

**Published:** 2018-04-13

**Authors:** Luigi La Spada, Lucio Vegni

**Affiliations:** 1 School of Computing, Electronics and Mathematics, Coventry University, Coventry CV1 5FB, UK; 2 Department of Engineering, University of Roma Tre, Via Vito Volterra 62, 00146 Rome, Italy; lucio.vegni@uniroma3.it

**Keywords:** metamaterials, nanoparticles, modeling, design, sensors, cancer detection, glucose measurements, medical diagnostics

## Abstract

A modeling and design approach is proposed for nanoparticle-based electromagnetic devices. First, the structure properties were analytically studied using Maxwell’s equations. The method provides us a robust link between nanoparticles electromagnetic response (amplitude and phase) and their geometrical characteristics (shape, geometry, and dimensions). Secondly, new designs based on “metamaterial” concept are proposed, demonstrating great performances in terms of wide-angle range functionality and multi/wide behavior, compared to conventional devices working at the same frequencies. The approach offers potential applications to build-up new advanced platforms for sensing and medical diagnostics. Therefore, in the final part of the article, some practical examples are reported such as cancer detection, water content measurements, chemical analysis, glucose concentration measurements and blood diseases monitoring.

## 1. Introduction

One of the most interesting physical phenomenon, recently used in advanced sensing and medical diagnostics, is the so-called Surface Plasmon Resonance (SPR). It arises from the coupling between the impinging electromagnetic wave and the electrons oscillation along the considered (metal) surface [1]. Sensors based on SPR use the well-established Kretschmann configuration [2]. Advantages for using it in sensing and diagnostics are mainly: easy fabrication process and fast response [3,4,5]. To further enhance the structure performances, several configurations have been explored during the past years such as dielectric [6] and/or metallic [7] layers, nano-structures [8,9], and thin films [10]. Recently, with the advent of fiber-optics technology, more sophisticated and miniaturized structures have been developed [11] with high focusing properties [12] and enhanced sensitivity [13,14]. Although fiber optics possess lower resolution and high sensitivity to external disturbances [15], they show a broader (spatial/frequency) bandwidth compared to the Kretschmann configuration. Thanks to recent and advanced fabrication techniques, new structures have been developed such long period [16] or Bragg gratings [17], nanometer devices [18,19], discontinuities [20,21] and new materials [22,23], presenting enhanced sharp resonances and improved performances. A peculiar type of materials that has been exploited in the past as biosensors is the so-called metamaterial. The basic design consists in array of electrically small particles (inclusions) embedded into a dielectric host material. They can be fabricated with 3D structures [24] or in thin-film versions (meta-surface) [25]. Typically, metamaterials possess newly engineered unusual electromagnetic characteristics not easily found in nature, primarily dependent on both inclusions properties and host material [26]. An example is represented by Split-Ring-Resonators (SRR), typically used to detect biomolecular bindings [27,28], chemical/biological compounds [29,30,31], and quantitative analysis [32]. A peculiar class of meta-materials used as plasmonic biosensor platforms have recently been developed: multilayer metal-dielectric metamaterials with Hyperbolic Dispersion (HD) [33]. Such materials demonstrate broadband and high sensitivity from near-infrared to visible frequencies. The main advantage of such structures is that the resonance position can be tuned among such range by simply changing both the HD properties and the diffraction grating parameters. Such structures offer promising opportunities to design new sensors to selective detect both higher and lower molecular concentrations in diluted solutions, crucial features for new generation lab-on-a-chip sensors [34]. 

Moreover, the new advanced particle synthesis technologies gave us the possibility to excite the SPR at nanoscale [35]. When the nanoparticle is illuminated by an appropriate incident electromagnetic wave, electrons oscillate and generate strong waves along the particle surface leading to strong field enhancement in its near field zone [36,37]: Localized-SPR (LSPR). LSPR characteristics and applications in sensing depend on different factors such as nanoparticle size, shape, composition and the dielectric constant of the local environment, inter-particle distances and the polarization of the incident light [38,39]. The LSPRs of metal nanoparticles (mainly silver and gold) happen in the visible and near infrared frequency regime, rendering them suitable as optical transducers/sensors or devices [40,41,42,43], capable of detecting a wide variety of targets, such as molecules, cells, cell compartments or viruses [44,45,46,47]. 

Nanoparticles geometry and composition strongly influence on the dielectric properties and thus affect the light-matter interaction in terms of possible plasmon modes and energy. Such effects are applied for the detection of the prostate specific antigen (PSA), which is important in the early diagnosis of cancer [48], demonstrating the possibility to detect minute concentrations of target proteins in complex matrices such as body fluids [49].

Changes in the surrounding environment can induce frequency shifts: processes such as molecular adsorption and chemical reactions can be monitored through the evolution of LSPR position [50]. Existing LSPR sensors can detect static and dynamic events in real time such as gas detection [51], protein–ligand and/or antibody–protein interactions [52], DNA hybridization [53], and protein changes [54]. 

One of the most important effects of LSPRs is the related strong electromagnetic near-field enhancement (FE), up to several orders of magnitude and spatially localized at nanometer scale. This gives the possibility to enhance also other molecular detection methods like Surface-enhanced Raman scattering (SERS) and Surface-enhanced fluorescence (SEF) [55,56,57,58].

The combination of the resonant effect and the additional enhancement of the field plays a crucial role in sensing technology [13], offering interesting advantages for sensitive and label-free purposes [14,59]:

- a significant reduction in the structure size and an improvement in resolution and sensitivity to enable the detection of small changes in the dielectric environment [60,61];
- tune the response (resonant frequency, amplitude and bandwidth) by tailoring the geometrical parameters to coincide with the absorption peaks of a well selected organic group [62,63];
- increase system performances [64], wireless measurements [65], material characteristics analysis [66] and therapy [67,68,69,70,71]; and
- avoid the employment of markers and eliminate artefacts caused by their use [72].

Despite all such examples present in the literature, they are confined to specific geometries, shapes and design frequencies. A general approach able to link all the steps from modeling to manufacturing is still missing: in this work, we developed a method to design arbitrary shape nanoparticle structures for specific required applications, focusing our attention on advanced sensing and medical diagnostics. 

This paper is structured as follows: First, a generic analytical model is derived in terms of (electrical/magnetic) polarizability to describe the scattering and absorption properties of the nanoparticles. Then, by exploiting such models and modifying the geometry/shape of traditional existing structures, new design examples are reported. Finally, some practical devices for sensing and medical diagnostics are shown.

## 2. Nanoparticles Modeling

**Figure 1:**
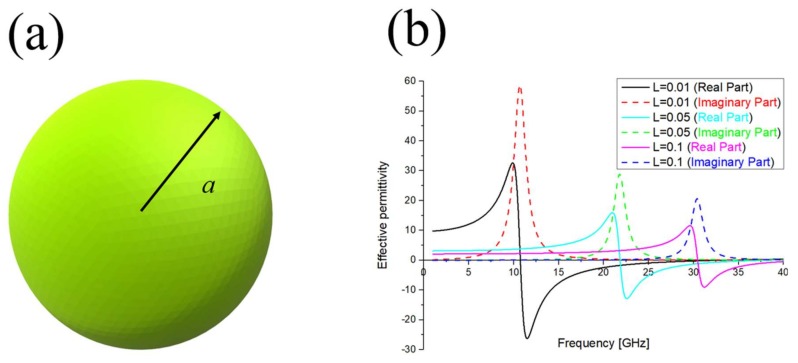
(**a**) Examples of spherical nanoparticle; (**b**) Variation of a generic nanoparticle dielectric permittivity (real and imaginary part) as a function of its depolarization factor *L*.

LSPR can be observed in metals, semiconductors or alloys possessing large and small values in the negative real and imaginary part, respectively, of the dielectric electric permittivity. Its absorption and scattering characteristics are determined by the geometry of the nanoparticles used (size and shape), their composition (metal, dielectric) and the refractive index of the surrounding environment [73]. Let us take as an example the spherical particle depicted in Figure 1a. To obtain the explicit form of the electromagnetic field distribution, some assumptions should be done [74]:

- The particle size is much smaller than the incident wavelength present in the surrounding medium: the wave phase can be considered nearly constant over the particle volume. We are under the so-called quasi static approximation.
- Both particle and surrounding material are considered homogeneous, isotropic and non-absorbing media.

Let us excite the structure by using an electromagnetic wave with the electric field parallel (and the propagation vector *k* perpendicular) to its principal axis. Under the quasi-static approximation, it is possible to relate the nanoparticle macroscopic electromagnetic characteristics (in terms of permittivity and/or permeability) to its microscopic behavior (electric/magnetic polarizability). To this regard, the scalar component of the dyadic polarizability (α*_x_*_,*y*,*z*_) of an arbitrary shape particle can be written as [75]:

(1) $$\alpha_{x,y,z}=V\varepsilon_{e}\frac{\varepsilon_{i}-\varepsilon_{e}}{\varepsilon_{e}+L_{x,y,z}\left(\varepsilon_{i}-\varepsilon_{e}\right)}$$

where *V* is the particle volume, *ε_e_* is the electric permittivity of the surrounding dielectric environment, *ε_i_* is the particle electric permittivity and *L_x,y,z_* is the depolarization factor along the coordinates *x*, *y*, *z*. The absorption (*C_abs_*) and scattering (*C_sca_*) cross-section expressions are [76]:

(2a) Cabs=kIm(α)

(2b) $$C_{sca}=\frac{k^{4}}{6\pi }{\left|\alpha \right|}^{2}$$

where *k* = 2*πn*/*λ* is the wave number, *λ* is the wave length and *n* is the refractive index of the surrounding dielectric environment. Similar results can be obtained if the inclusion is considered real: its permittivity should be written as *ε_i_* = *ε_r_* + *jε_i_*. From Equation (1), it is evident that the polarizability *α* can be maximized, in other words the nanoparticle is at its resonance, when:

(3) $$\{\begin{array}{c} Re\left[\varepsilon_{e}+L\left(\varepsilon_{i}-\varepsilon_{e}\right)\right]=0\\ Im\left[\varepsilon_{e}+L\left(\varepsilon_{i}-\varepsilon_{e}\right)\right]=0 \end{array}$$

Equation (3) confirms that absorption and scattering characteristics strongly depends on the geometry of the structure (*L*), the inclusion properties (*ε_i_*), and the surrounding environment refractive index (*ε_e_*), as also shown in Figure 1b. 

In Section 3, we see how to exploit geometrical and material characteristics to tailor and design nanoparticle response in terms of scattering/absorption properties. In Section 4, we exploit the dependence of the surrounding medium refractive index to develop practical applications in sensing and medical diagnostics.

## 3. Nanoparticles Design Approaches

LSPR properties and their design are currently the main subjects of sensing and diagnostic research [77]. The first example of LSPR device was a sphere-shaped nanoparticle arranged in a planar array configuration. Due to its simplicity and easiness in its realization, it has been extensively theoretically studied [78] and implemented in different application fields [79]: telecommunications, forensics, military, sensing, and medical diagnostics. One of the main drawback of spherical particles is the field strength and distribution: in general, too weak and not well-confined. Therefore, during the years further structure geometries and field enhancement techniques have been developed. In the following, we explore some that are useful to design practical devices for sensing and medical diagnostics.

### 3.1. Inter-Coupling Effect

**Figure 2:**
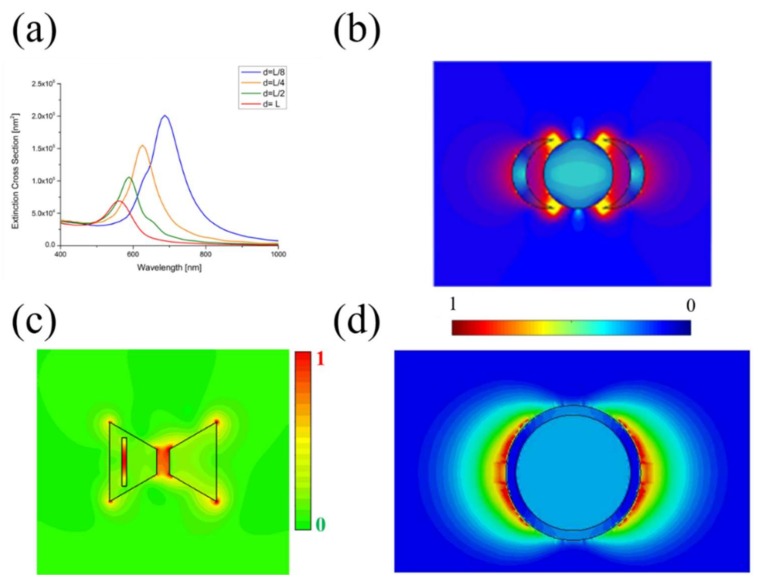
(**a**) Extension cross-section spectra for different inter-particle distance. Near electric field distribution for: (**b**) Multi-polar resonant structure; (**c**) Modified bow-tie particle; and (**d**) The core/shell particles at the resonant PMMA-Graphene plasma wavelength (572 nm).

Classical antenna theory array [80] inspired this LSPR enhancement technique. The array is typically intended as a set of multiple connected scatterers which work together as a single one, to transmit/receive signals with a specific phase relationship. In the case of nanoparticles array, the signal radiated/received by each individual scatter combine and superpose, adding together to enhance the power in desired directions (focusing effect), and/or cancelling each other to reduce such power in other directions. The focusing effects arise from the charge induction between the two particles, interacting stronger as they get closer to each other. From the mathematical point of view, the number and position of the elements can be arbitrarily varied to tailor the response *F(θ,φ)* at will [81]:

(4) $$F\left(\theta ,\varphi \right)=\overset{N}{\underset{i=1}{\sum }}\left(a_{i}+\overset{N}{\underset{\begin{array}{c} k=1\\ k\neq i \end{array}}{\sum }}C_{ik}\right)e^{jk_{0}\left(r•\hat{r}\right)}$$

where *a_i_* is the excitation coefficient of the single array element and *c_ik_* is the coupled coefficient between *i* and *k*-th array element. The coefficients *c_ik_* depend on the inter-particle distance. From Equation (4), it is clear when the distance between particles increases, the coefficients decrease and the near electric field distribution is weaker (Figure 2a). An optimal near-field enhancement is achieved when the inter-particle distance is equal or less than its physical dimensions.

### 3.2. Multipolar Resonance

For the simple geometries existing in the literature (sphere and cube), classical approaches exploit only the dipolar polarizability resonance to design appropriate particle response [78,79]. On the other hand, more advanced nanostructures can use high-order (multipolar) resonance modes, by increasing their aspect ratio. Both transverse and longitudinal modes are separated, and they appear as two distinct plasmon resonant peaks in the response spectrum [82,83]: localized and confined electromagnetic field enhancement, called “hot spot”, are present providing great sensitivity and high-resolution performances. Multipolar resonances can be introduced in several nanostructures such as the nanocrescent [84,85]. As reported in Figure 2b, the field intensity is larger at the edges of the nanocrescent and strongly dependent on the wave polarization, leading to two depolarization factors, transverse and longitudinal, respectively:

(5) $$\begin{array}{l} L_{trans}=\frac{1}{\sqrt{1+\frac{16a^{2}}{{\left(4a+h\right)}^{2}}}}\\ L_{long}=\frac{1}{2}\left(1-L_{trans}\right) \end{array}$$

where *a* is the particle radius, *h* is its height and *L_trans_/L_long_* is the transverse and longitudinal depolarization factor, respectively.

The two depolarization factors result from the difference of accumulated electrons at the sharp edges: if the wave is horizontally polarized (*L_trans_*), the electrons are equally separated to both ends of the nanocrescent; on the other hand, if the light is vertically polarized (*L_long_*), electrons are enabled to freely move to sharp edges of the particle, with consequently higher field intensity.

### 3.3. Plasmon Hybridization

Besides spherical particles, researchers focused their attention on cylindrical symmetry particles called *nanorods* for the following reasons: high sensitivity and resolution to any refractive index changes, huge LSPR strength enhancement caused by its needlelike shape, easiness in fabrication [86], and the possibility to tune their absorption and scattering efficiency by varying the aspect ratio [87]. Such properties derive from a phenomenon called “plasmon hybridization” [88]: nanorods can exhibit both longitudinal and transverse plasmon modes (polarizability components). Each mode and the related properties will depend on the particle orientation respect to the incident wave direction as well as the axis geometrical dimensions [89].

By exploiting plasmon hybridization, in [90], an analytical model for modified nanorods was proposed. Their electromagnetic properties, in terms of extinction cross section (absorption and scattering) for both longitudinal and transverse modes excitation, can be evaluated by using the following general design formula:

(6) $$\frac{a_{x,y,z}}{V_{2}\varepsilon_{m}}=\frac{\left(\varepsilon_{2}-\varepsilon_{m}\right)\left(L_{1}\varepsilon_{1}-\left(L_{1}-1\right)\varepsilon_{2}\right)+\beta \left(\varepsilon_{2}-\varepsilon_{1}\right)\left(\left(L_{2}-1\right)\varepsilon_{2}-L_{2}\varepsilon_{m}\right)}{L_{2}\beta \left(L_{2}-1\right)\left(\varepsilon_{2}-\varepsilon_{1}\right)\left(\varepsilon_{2}-\varepsilon_{m}\right)-\left(\left(L_{1}-1\right)\varepsilon_{2}-L_{1}\varepsilon_{1}\right)\left(L_{2}\varepsilon_{2}-\left(L_{2}-1\right)\varepsilon_{m}\right)}$$

being *V_i_* the volumes, *L_i_* the depolarization factors, *ε_i_* the electric permittivity, *β = V*_1_*/V*_2_ the filling fraction, and *i =* 1 (core), 2 (shell), *m* (environment). The proposed modified particles (core/shell structure embedded in a dielectric environment) allow us to add supplemental degrees of freedom (the shell thickness and the core material) in the design of their optical response, compared to classical single-phase particles.

Plasmonic hybridization has also been extensively used for other nanostructures such as nanorings, nanoshells [91], nanodisks [92], trimers [93], and oligomers [94]. 

### 3.4. Fano Resonance and Symmetry Breaking

Fano resonance in nanoparticles can arise from the coupling/interference effects between bright and dark plasmon modes: it is the direct consequence of the structure *symmetry breaking* [95]. By using such a method, in [96], the electromagnetic properties of modified bow-tie nanoparticles were investigated. They consist of a pair of opposing (metallic) truncated 2D triangles with a dielectric hole accurately engraved:

(7) $$L_{bow-tie}=\frac{3}{\pi }arctan\left[\frac{a\cdot e}{\left(b+\frac{d}{2}\right)\sqrt{a^{2}+4{\left(b+\frac{d}{2}\right)}^{2}+e^{2}}}\right]$$

where *L_bow-tie_* is the entire particle depolarization factor, *a* is the side length, *b* is the inner length, *d* is the gap width and *e* is the gap length.

Under such circumstances, both multi/wide-band behavior and high electric field localization can be obtained, as shown in Figure 2c. This represent a huge advantage in using such a structure for sensing and diagnostics. From Equation (7), it is possible to see how we can easily tune the nanostructure resonances by simply changing its geometrical and electromagnetic characteristics. This is a crucial step to design specific devices whose resonant frequencies coincide with the spectral absorption characteristics of the sample under study. Moreover, the presence of multiple frequencies and additional hot spots improve both the sensitivity and the resolution performances of the entire device.

### 3.5. Plasmonics Effects and Near-Zero-Index (NZI) Materials

Recent studies focused their attention on a specific class of advanced materials entitled near-zero-index (NZI) and on their peculiar electromagnetic properties. Such materials possess low (mostly near zero) values of the constitutive parameters (permittivity/permeability), leading to interesting applications such as wave phase-front manipulation [97,98], directive radiating elements [99], optical nanocircuits [100], electromagnetic confinement [101], transmission enhancement [102], anomalous tunneling effects [103,104,105], field focusing [106], cloaking [107,108] and sensing systems improvement [109,110,111]. Following the recent success of NZI materials, in [112], a new kind of nanoparticle by combining both plasmonic and near-zero effects was proposed. The structure consists of core-shell spherical inclusions: PMMA-Graphene core and metallic (gold) shell. In the case of an NZI media, the starting effective refractive index is near zero and the relative change caused by the modification of the background material permittivity will be very large. Therefore, the resulting refractive index will be approximately equal to the permittivity change due to the background material [113,114]. In this kind of structure two phenomena are both existing: the LSPR effect responsible for the electric field enhancement around the particle and the NZI effect that leads the electric field lines outside the core particle (the effective structure permittivity has a very low value at the resonant frequency). Both phenomena ensure a much stronger field interaction with the surrounding material, restricted in a small area, compared to the traditional cases (Figure 2d).

### 3.6. Electromagnetic Nanoparticle Sensitivity and Applications to Single Molecule Detection

Electromagnetic nanoparticles and nanostructures have been exploited for amplification of the sensitivity of existing sensors, as well as for the design of new types of sensors. Biochemical and medical applications gain from LSPR phenomenon in terms of disease diagnosis, in vivo detection, imaging, cell tracking, and monitoring disease pathogenesis or therapy progress [115,116,117]. To provide an insight on LSPR-nanoparticles sensitivity (*S = Δf/Δn* [nm/RIU]), we present in Table 1 a comparison of the common electromagnetic technology sensors and the different approaches described before.

Recently, the detection of a single molecule became the ultimate challenge in sensing applications. This goal can be achieved in different ways, by exploiting the enhancing methods reported in the previous paragraphs. When two individual spherical nanoparticles are close to each other, they become coupled due to the interaction of their dipoles forming a *dimer*. The coupled *dimer* leads to an additional resonance at a lower energy, strongly dependent on the gap size between the particles. In [131,132], such a principle was used to detect DNA at very small concentrations. 

In complex structures such as nanostars and nanorings, the LSPR modes are the result of plasmon hybridization of individual modes [133,134]. Because of the long wavelength (low frequency) of these LSPR modes, the refractive index sensitivity toward environmental changes is expected to be high. A representative example is an H_2_ sensor, crucial in new technological energy storage applications such as fuel cells [135]. Different technologies have been implemented for H_2_ sensing such as: Au discs decorated with Pd nanoparticles and separated by a SiO_2_ dielectric spacer [136,137], single shell-isolated nanoparticles [138] or an Au antenna–Pd particle system [139].

The detection of single molecules is reliable only when the sensing volume is comparable to the size range of the molecule. Therefore, direct sensing only works for large molecules such proteins. For small molecules (such as aminothiophenol (ATP)) the LSPR response must be enhanced by plasmon coupling. Such a technique has been exploited by using Au nanoparticles to detect a complementary DNA strand in real-time [140], and by using single nanorods to observe single protein binding events of the blood plasma protein fibronectin with high temporal resolution [141]. 

The possibility to resolve events at high resolution can make all the enhanced methods useful for the study of protein adsorption processes, and to monitor matter kinetics and dynamics at the single molecule level.

## 4. Nanoparticles Sensors and Applications in Medical Diagnostics

Until now, a general overview on how to model and design nanoparticles has been reported. In this paragraph, we use and apply such design methods for sensing and diagnostic applications. The main idea is based on detecting small local changes in the refractive index of biological events. When the nanoparticle is small, it is highly sensitive and possesses great spatial resolution for single molecules detection [142] purposes. At the same time, despite this, its size should be carefully chosen to ensure that the signal intensity is sufficiently high for a significant frequency shift in the spectrum.

**Figure 3:**
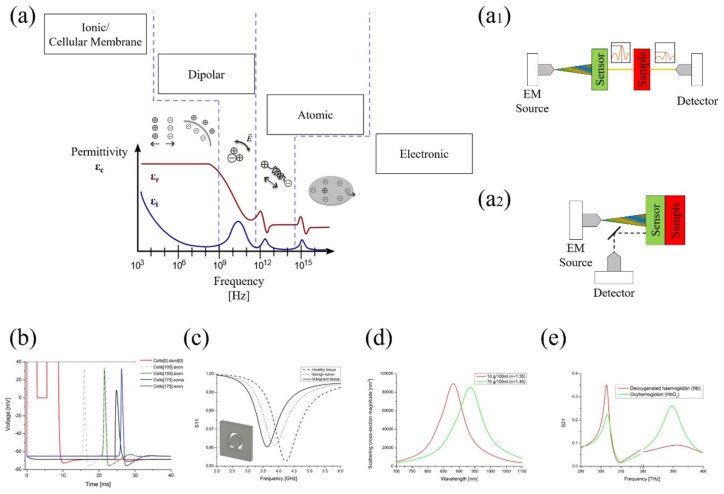
(**a**) Biological material permittivity spectrum over frequency: *ε_r_* and *ε_i_* denote the real and the imaginary part of the permittivity, respectively. Various processes arise as a function of frequency: ionic and dipolar relaxation, atomic and electronic resonances at higher energies. There are two kind of sensing platforms: (**a_1_**) Direct contact and (**a_2_**) Distance measurements. (**b**) Pulse propagation along dendride 0 of neuron 0 (red line), axon of neuron 100 (gray dashed line), axon of neuron 150 (green line), target neuron soma (black line) and target neuron axon (blue line) [71]; (**c**) Resonant frequency shift among healthy tissue (step-line), benign tumor (dot) and malignant tissue (straight line); (**d**) Extinction cross-section spectra for glucose concentration measurements; and (**e**) transmission spectra for Oxyhemoglobin and Deoxygenated hemoglobin at reference absorption frequencies [72].

Dielectric properties of materials can be described by their dispersive complex (real and imaginary part) dielectric permittivity *ε_c_* expressed as: *ε_c_* = *ε_r_* + *jε_i_* (being *ε_r_* the relative permittivity of the material and *ε_i_* the out-of-phase loss factor) or the associated σ total conductivity as a function of frequency *ω* of the applied field. In general, tissue dielectric properties and their frequency response are the results of the interaction between the electromagnetic radiation and their constituents at cellular and molecular level, as shown in Figure 3a. Such an interaction can be described by two different mechanisms that influence the shape of the permittivity:

- Dielectric relaxation is the result of the movement of dipoles (dipole relaxation) and electric charges (ionic relaxation) due to an applied alternating field [143,144,145]. In particular:
  ○ **Ionic relaxation** comprises ionic conductivity and interfacial (space) charge relaxation. Ionic conductivity predominates at low frequencies and introduces only losses to the system. Interfacial relaxation occurs when charge carriers are trapped at interfaces of heterogeneous systems.
  ○ **Dipole relaxation** originates from permanent and induced dipoles aligning to the electric field. The time needed for dipoles to relax (relaxation time/frequency) is determined by the local viscosity. Dipole relaxation is heavily dependent on temperature, pressure and tissue constituents. In tissues, such a dispersion phenomenon is due mainly to the polarization of cellular membranes which act as barriers to the flow of ions between the intra and extra cellular media. Other contributions to such a dispersion come from the polarization of protein/organic macromolecules and polarization of water molecules.
- At higher frequencies and smaller scales, resonance processes arise from the rotations/vibrations of atoms, ions, or electrons. These processes are observed near their characteristic absorption frequencies [146]. In particular:
  ○ **Atomic polarization** is observed when the nucleus of the atom reorients in response to the electric field. It is intrinsic to the atom nature and it is a consequence of the applied field. Atomic polarization is usually small compared to electronic polarization.
  ○ **Electronic polarization** occurs when the electric field displaces the *electron* density relative to the nucleus it surrounds. This displacement occurs due to the equilibrium between restoration and electric forces.

Each dielectric mechanism is centered around its characteristic frequency: relaxation mechanisms are relatively slow and are usually observed in the frequency range 10^2^–10^10^ Hz, compared to resonant electronic transitions or molecular vibrations, which usually have frequencies above 10^12^ Hz.

At low frequency where we are interested in ionic relaxation phenomena, we need appropriate process characterization tools, mainly based on conductivity/impedance measurements (Figure 3b). Highly-sensitive detection of drug/neurotransmitters was shown in [147] using a 3D graphene biosensor for the detection of dopamine reaching a limit of detection of 1 nM. In [148], an in-situ detection of an antipsychotic drug in serum for therapy monitoring was demonstrated. These methodologies did not analyze the intrinsic relation between the drug and the neurotransmitter, crucial information in the drug discovery process. Electrochemical Impedance Spectroscopy (EIS) can fill this gap [149]. Such a technique reveals the response of an electrochemical system (like a cell) to an applied potential. The frequency dependence of the system impedance can reveal the underlying chemical processes. In [150], the authors proposed a novel analysis strategy demonstrating the great potential offered by label-free biosensors as integratable and efficient tools for antipsychotic drug screening and analysis.

Regarding the microwave regime, diseases typically induce structural, biochemical and mechanical changes in tissues, leading to significantly different permittivity values. The main aim of an electromagnetic biosensor is to reveal such differences, by correlating the substance dielectric properties to its resonant properties. The output signal will have the resonant characteristics (resonance position, magnitude and bandwidth) depending on such modifications. For this reason, the microwave frequency range is extremely useful for the detection of cancer and blood diseases. To detect such alterations, two kind of sensing platforms can be used [151]: 

(1) Direct contact measurements: The device itself possess a specific response in terms of frequency position, amplitude and phase. Once the tissue to study is placed in contact, the overall device characteristics change as a function of the tissue properties (Figure 3a_1_). From the frequency position of the resonant dip, it is possible to discern the substance that we are looking for.

The presence of water in a biological tissue produces changes in its permittivity and conductivity values [143,144,145]. A tumor, in fact, has a significantly higher water content compared to normal tissues [152]. Therefore, the permittivity ε and the conductivity σ of the tumor are higher than those of a normal tissue [153]. This is valid not only at microwaves but also at mm/THz frequencies. Such property turns out to be a useful tool for tumors detection. Some examples, in microwave and THz for the detection of a cancerous tissue and different cancer stages are presented by Smith et al. [66] and Johnson [154], respectively (Figure 3c).

(2) Distance measurements: The sensor is placed at a specific distance from the biological sample. Unlike the previous situation, the changes in the biological tissue are detected in the transmission coefficient magnitude and amplitude width, while the resonant wavelength position does not change (Figure 3a_2_). From the resonance magnitude and bandwidth, we reveal the sample concentration.

The permittivity of water solutions increases with the increasing of the chemical species concentration: it would be possible to sense the presence of either organic or inorganic compounds in a water solution, with possible applications in food and medical diagnostics. In this way, nanoparticles can be used for quantitative analysis of many substances [155]: alcohol content, sugar, and acidity; and extractable substances with and without sugar, such as glucose concentration in aqueous solution.

Organic and inorganic compounds absorb in specific spectral regions. This property gives each material a unique signature in the electromagnetic spectrum, depending on the related molecular structure [156]. Thus, the specificity and uniqueness of such spectra can be used for the recognition of the biological tissue under test. Therefore, it is necessary to irradiate the sample in a selective way, to excite only the chemical groups of our interest, leaving unaltered the other absorption bands of other chemical bonds. This method can be used for cancer tissue diagnostics [157]: at infrared frequencies, the major differences between normal and cancer tissue are at their structural and molecular level. Proteins and lipids have spectra in malignant tumors different from those present in benign tumors or in normal tissues: such three groups of biological samples possess different absorption peaks in the spectrum [158]. Starting from such differences, it is possible to recognize if the sample belongs to a healthy tissue or to a cancerous one [159], its water content [160] or evaluate the oxygen saturation in blood samples (Figure 3d). 

At higher frequencies, plasmonic sensors such as the well-known surface plasmon resonance (SPR) systems have been applied for direct dopamine detection, achieving sensitivities in the pM range [161,162]. Glycerol measurements in aqueous solution have recently captured researchers’ attention in several application fields such as biomedical engineering, medicine and biofuels [163]. Its measurement is useful to evaluate various parameters such for example the possibility to enhance optical clearing of skin [164]. Glycerol is also important in industrial fermentation processes [165]. Its measurement is not a simple task since its permittivity varies not too much by changing its chemical concentration. From this arises the necessity of using extremely sensitive sensing systems [96,151]. Accurately designed LSPR sensors [154] allow the detection of glycerol concentration when different concentrations are considered [166,167,168,169] (Figure 3e).

Recently, in [150], plasmonic nanohole arrays have been used as biosensor, based on the Extraordinary Optical Transmission (EOT) phenomenon [170,171]. This light transmission enhancement is attributed to the coupling between the incident light with the grating surface. It is characterized by the appearance of peaks and dips in the transmission spectrum. Such modes are so sensitive that any changes in the refractive index (surrounding the nanoholes) will induce a shift in the EOT peak wavelength [172,173]. This sensing principle enables not only label-free and real-time detection but also multiple analysis of different drugs simultaneously, reducing time and costs [174]. In addition, EOT can be achieved by normal light incidence and it is compatible with the use of common existing electronics such as light emitting diodes, Complementary metal–oxide–semiconductor (CMOS)-based imaging systems, and advance microscopes [175], allowing miniaturization and wide field-of-view [170,176]. In terms of sensitivity, it is possible to reach limits of detection at the nM level.

Beyond optical frequencies other techniques are implemented, making use of radioactive or fluorescent labels to monitor the response of sensor-analyte interaction [177,178]. However, labeling is not only a long and costly procedure, but it also generally creates several problems due to the presence of false positives and/or false negative. Therefore, label-free and sensitive screening methods are required: LSPR biosensors provide unique solutions as they can give information in a fast and easy manner [179,180,181,182,183,184,185,186,187,188]. 

## 5. Current Challenges and Future Perspectives

Over the last few years, electromagnetic nanoparticles played a significant role in the development of advanced medical devices and in different application fields. However, at the same time, there are still several challenges and controversies. 

A fully integrated biosensor capable of performing multiplexed and label-free detection at physiologically concentrations has not been accomplished until now [189]. In other words, the ability to detect multiple samples simultaneously, with enough sensitivity, and directly in human fluids [190,191] is a technology in its infancy. From a clinical and technological point of view, existing platforms still face the following issues: long test time, extensive sample preprocessing and complexity of current systems, temperature dependence, wide dynamic linear range, sensitivity and portability. The development of recent electromagnetic nanotechnologies has addressed some of these requirements. For example, mass-sensitive piezoelectric and microcantilever-based systems (by using surface oscillations/changes due to surface stress) have simplified sample-preparation steps [192,193]. Electrical detection systems (such as electrochemical sensors and/or electrodes) can provide affordable, simple, and straightforward measurements [194,195,196,197]. Furthermore, plasmonic-based platforms have minimized system complexity and demonstrated quantitative and sensitive measurements [198,199,200,201,202,203,204,205,206,207,208,209,210].

However, such advances have only reduced some of the above issues, as expected other critical challenges remain not addressed. Therefore, significant efforts have been focused to improve them on a single platform, by using the so-called “Nanoplasmonic Electrical field-Enhanced Resonating Devices” (NE^2^RD). Such a structure consists in a platform that senses, detects, and quantifies a wide set of biological targets (such as proteins, drugs, viruses, bacteria, and eukaryotic cells), allowing timely monitoring of disease progression and response to therapies [211].

The in vitro screening and analysis of drug interactions with the cellular receptors is essential not only to determine the mechanism of action, but also to quantify the affinity and obtain preliminary dose–response curves [212]. Therefore, it is necessary to establish new rigorous methods that enable accurate analysis and efficient screening, reducing time and costs in the development process.

Potential chronic and acute toxic effects: nanoparticles may be attached to the surface of biological membranes by adsorption or electrostatic interactions, and they can cause damage to cells by producing reactive oxygen species, leading to protein denaturation, lipid peroxidation, DNA damage, and cell death [213,214,215,216]. It is necessary to carry out a detailed toxicity study to ensure safety prior to further applications in humans: nanoparticles need to be understood more deeply before their potential application in tumor therapy [217].

Thanks to their extraordinary tunable properties, we can expect to produce several breakthroughs and pave new ways to diagnose several diseases and use them for tumor therapy, based on their size, biocompatibility, chemistry, and toxicity in biological systems. It is highly expected that their application in tumor therapy will greatly improve current methods of detection, imaging, and therapy, while reducing the use (and toxicity) of traditional tumor treatments. The conversion of nanoparticles to routine clinical practice require a multidisciplinary approach and different expertise. In view of the recent significant research results and advances obtained, there is no doubt that humans will greatly benefit from nanoparticles in the very near future, especially in tumor therapy.

## 6. Conclusions

An overview of modeling and design methods and their main applications for nanoparticles-based sensors has been reported based on their electromagnetic properties. The considered structures consist of resonant inclusions arranged in array configuration, whose electromagnetic response (position, magnitude and amplitude width) can be modified through changes in the surrounding environment. 

Modeling and design such structures is important to understand how their geometrical factors (dimensions, shape, and volume fraction) and electromagnetic properties influence the overall device behavior. By exploiting different methods, we can manipulate and control the main nanoparticle properties for specific applications, with both improved sensitivity and high spectral resolution. 

The possibility to use them as platforms (from microwave to optics) for permittivity and absorption measurements in sensing and medical diagnostics has been reported. The shown design methods, with appropriate modifications, can be extended to any other application fields such as imaging, forensics, defense and security.

## Figures and Tables

**Table 1:** Sensitivity (nm/RIU) comparison for different LSPR technologies.

Structure		Frequency Range (nm)	Sensitivity (nm/RIU)
Kretschmann configuration [118,119]		400–900	7500–30 k
Fiber-Optic sensors [120]		400–1600	2000–9800
Nano-structured coupling [121,122]		600–2000	440–600
Nanoparticle-based sensors	Periodic array (Inter-Coupling effect) [123]	400–950	200–350
	Gold nano-ring (Multipolar Resonance) [124]	300–1800	650
	Pair/disk pair (Plasmon Hybridization) [125]	500–900	170 k
	Un-periodic array (Fano Resonance/Symmetry breaking) [126]	300–700	165
	Graphene Core-shell spheres (Plasmonic and NZI) [112]	520–870	421
Interferometer [127,128]		800–1550	250–4547
Meta-surfaces [129,130]		1400–1600	600
